# Family Functioning, Emotional Intelligence, and Values: Analysis of the Relationship with Aggressive Behavior in Adolescents

**DOI:** 10.3390/ijerph16030478

**Published:** 2019-02-06

**Authors:** María del Carmen Pérez-Fuentes, María del Mar Molero Jurado, Ana Belén Barragán Martín, José Jesús Gázquez Linares

**Affiliations:** 1 Department of Psychology, University of Almería, 04120 Almería, Spain; mmj130@ual.es (M.d.M.M.J.); abm410@ual.es (A.B.B.M.); jlinares@ual.es (J.J.G.L.); 2 Department of Psychology, Universidad Autónoma de Chile, Región Metropolitana, Providencia 7500000, Chile

**Keywords:** family functioning, aggressive behavior, emotional intelligence, adolescent values

## Abstract

Aggressive behavior in adolescence is influenced by a diversity of individual, family, and social variables. The purpose of this study was to analyze the relationship between family functioning, emotional intelligence, and personal values for development with different types of aggression, as well as to establish profiles with these variables according to the aggression. The study was carried out with a sample of 317 high school students aged 13 to 18 years old. The study showed that stress management (emotional intelligence), positive adolescent development, and family functioning predominated in nonaggressive subjects with higher scores than aggressors did. There was also a negative relationship between the different types of aggression and emotional intelligence, positive values, and family functioning. In addition, two different profiles were found. The first profile had less family functioning, interpersonal emotional intelligence, stress management, and fewer personal and social values than the second profile.

## 1. Introduction

Violent behavior among secondary students has been identified as a serious problem in education today [1], with a corresponding increase in the number of studies on this topic in the literature [2,3]. Recent studies in several countries have shown that the prevalence of such adolescent behavior has increased [4,5], and poses a risk both for students who do not show this kind of behavior and educational activities in the school [6]. In view of the many problems in this context, the quality of coexistence in the school must be known before actions can be proposed or resources created [7]. 

### 1.1. Violent Behavior in the School Environment

Both physical and verbal aggressive behavior patterns may be found in the school, and these may be direct or indirect. For example, direct physical aggression refers to hitting someone while stealing would be an example of indirect physical aggression [8]. Direct verbal aggression refers to the insults between the actors involved in such situations, while talking about someone behind their back is considered indirect verbal aggression [9].

Aggression may also be classified by the method used to cause harm by differentiating between form and function [10]. The form of the aggression may be overt, which is manifested as physical and verbal aggressive behavior, such as threatening and hitting, or relational, which damages social inclusion within a group by social exclusion [11]. Function includes reactive aggression, which is characterized by behavior for revenge or getting even, while proactive aggression does not require provocation [12]. Both types of aggression have been studied in adolescent populations and in school-age children. Thus, the study by Manring, Elledge, Swails, & Vernberg [13] in primary school showed reactive aggression as a longitudinal predictor of peer victimization, especially in girls. Similarly, individual factors are linked to relational aggression by girls, where it is an indicator of this type of problematic behavior [14]. There are also gender differences in the different types of aggression [15], where boys have higher percentages of reactive and proactive aggression than girls [16,17]. However, van Hazebroek, Olthof, and Goossens [18] found higher levels of reactive aggression in the group of boys than the group of girls, and no gender differences was found in proactive aggression. 

Victim and aggressor profiles have different types of aggression. Jara, Casas, and Ortega-Ruiz [19] suggested that aggressors show proactive aggressive behavior, while reactive aggression is present in victims. Studies on relational violence have found different results concerning the sex of the aggressors. Ettekal and Ladd [20] showed that females participate more in relational violence. However, in other studies, no significant difference was found between men and women. 

### 1.2. Variables Related to Aggressive Behavior

Adolescence is characterized by experimentation and sensation-seeking associated with impulsivity [21]. Aggression among youths is also linked to a series of individual, academic, family, and social factors [22]. Aggressive behaviors are related to personal and social values [23,24]. According to Jara et al. [19], social values are more important to aggressors than to victims, personal values are important to both agents, and individual values are stronger in aggressors than in victims. These authors also showed that there is a relationship between being a participant in aggressive behavior or not and social, personal, and individual values.

Likewise, there is a relationship between the different types of aggression and values for adolescent development, with a negative relationship between social and personal values and the different types of proactive and reactive aggression [25]. And in some studies, a positive relationship has been found between individualistic values and proactive aggression [26]. In turn, the family shows an impact on the internalization of the child’s values, which are all supported by empirical evidence [27,28,29,30].

Aggressive behaviors are related to emotional, social, and other variables [31]. The study by Zych, Beltrán-Catalán, Ortega-Ruiz, and Llorent [32] showed that bullies had low levels of social and emotional competence, while their victims had the same levels as the students who were not involved. The family also plays an essential role in the emotional competence of the child [27,30,33,34]. Studies on emotional intelligence in victims and aggressors have demonstrated that students who are victims have low emotional intelligence. This means they have less ability to cope with stressful situations, and the aggressors show low levels of emotional intelligence as well, with low emotional regulation and deficient stress management [35]. At the same time, some authors state that poor emotional regulation is a characteristic of reactive aggression [17,36]. 

Regarding the variables of the family environment, even during adolescence, the family plays a key role in several different domains of adolescent competence and adaptation [33,37]. Aggressive behavior has been found to be related to family functioning [38]. Parents are a source of influence on youth functioning [39,40] along with the group of peers, since conflictive settings, where conflict, criticism, insults, and lack of affection take precedence, may lead to aggressive behavior by not relating such behavior to its consequences [41]. According to studies done to date, parenting styles that employ physical and verbal aggression and hostile behavior are related to both the reactive and proactive functions of aggression. Thus, negative parenting and a dysfunctional environment are associated with proactive and reactive aggression [42]. 

In a recent study with Spanish adolescents [43], the family influence was analyzed in adolescents’ aggressive behaviors, antisocial behavior, school adjustment, and self-esteem. They were able to verify that the family was not only a protective factor against aggressive behaviors, but benefitted school adjustment and self-esteem. These, in turn, acted as protectors against antisocial behavior.

The review of the literature showed the relationship between types of aggression and variables such as emotional intelligence, values, and family functioning to be negative [44], personal and social values diminished [45], and a higher risk of family dysfunction [46].

At the present time, there are few studies profiling subjects by type of aggression [47], or the relationship of these types and emotional intelligence, family functioning, and values. 

### 1.3. The Study

The objective of this study was to analyze the relationship between emotional intelligence, development values, family functioning, and the various types of aggressive behavior. At the same time, we wanted to form profiles by aggression predictor variables for the different types of aggression. 

In view of previous studies, the following specific hypotheses were posed: (1) student aggressors score higher on all types of aggression than non-aggressors, (2) emotional intelligence, values, and family functioning are predictor variables of the appearance of aggressive behavior in the study sample.

## 2. Materials and Methods

### 2.1. Participants

A cross-sectional study was done with random cluster sampling. The sample consisted of a total of 317 students aged 13 to 18 years old (*M* = 14.93, *SD* = 1.06) at two high schools in the province of Almeria (Spain). The group of boys made up 50.8% (*n* = 161) with a mean age of 14.85 (*SD* = 1.00) and girls 49.2% (*n* = 156) with a mean age of 15.01 (*SD* = 1.11). The sample was distributed over two grades, 61.5% (*n* = 195) in the third year of high school and 38.5% (*n* = 122) in the fourth year.

### 2.2. Instruments

An ad hoc questionnaire for sociodemographic data (age, sex, grade) and questions on student involvement in peer violence at school (“Have you ever experienced episodes of violence by other students?” “Have you ever used violence against the other students?” “Have you ever seen violence against other students?” “Did you intervene when you saw someone using violence against other students?”).

Adolescent aggression was assessed with the *Peer Conflict Scale* (PCS) [48]. The Spanish adaptation from Pérez-Fuentes et al. [49] was used. This scale evaluates the overt and relational forms and reactive and proactive functions of aggression. It consists of 40 items, with answers rated on a four-point Likert-type scale (where 0 is not at all true and 3 is definitely true). In this study, the reliability for each scale was 0.81 for overt proactive aggression (item sample, “I start fights to get what I want”), 0.85 for overt reactive aggression (item sample, “When someone hurts me, I just get into a fight”), 0.81 for Relational proactive aggression (item sample, “I like to make fun of others”), and 0.78 for Reactive relational aggression (item sample, “I spread rumors and lies about others when they do something bad to me”), and, for the overall scale, reliability was *α* = 0.92.

Family Functioning was evaluated with the *Family Functioning Scale* (APGAR) [50]. The Spanish adaptation of the original version [51] was used. This scale consists of five items, which evaluate Adaptability, Growth, Partnership, Affection and Resolve (“Are important decisions taken together at home?”), with three answer choices (0 = hardly ever, 1 = some of the time and 2 = almost always). There are also three function categories, highly dysfunctional (0 to 3), moderately dysfunctional (4 to 6), and functional (6 or more). In this study, the Cronbach’s alpha was of 0.75. Regarding the validity of the scale, it has been discussed by authors such as Gardner et al. [52].

Emotional Intelligence was evaluated with the *Brief Emotional Intelligence Inventory for Senior Citizens* (EQ-I-M20) [53]. The adaptation by Pérez-Fuentes, Gázquez, Mercader, and y Molero [54] was validated and scaled in an adult Spanish population. This inventory is comprised of 20 items, which are distributed in five factors including Intrapersonal (It’s easy for me to tell people how I feel), Interpersonal (“I know how other people feel”), Stress Management (“I find it hard to control my rage”), Adaptability (“I can solve problems in different ways”), and Mood (“I feel sure of myself”), which was answered on a four-point Likert-type scale. For this sample, the instrument’s internal consistency was 0.78, and, for each of the subscales, reliability was Intrapersonal *α* = 0.77, Interpersonal *α* = 0.67, Stress Management *α* = 0.76, Adaptability *α* = 0.46, and Mood *α* = 0.83. 

Values for Positive Adolescent Development were evaluated with the *Scale of Values for Positive Adolescent Development* (EV-DPA) [55]. This consists of 24 items, which evaluate the importance that values are given by youths for their positive development. The answers are rated on a scale from 1 to 7, where 1 is “not important at all” and 7 is “the most important.” The scale is comprised of three dimensions: Social values (Defend the rights of others), Personal values (Be honest with others), and Individualist values (Receive praise from other persons), which had the following reliability: Social values *α* = 0.88, Personal values *α* = 0.83, and Individualist values *α* = 0.79, and the overall scale had a Cronbach’s alpha of 0.91.

### 2.3. Procedure

First, the high school principals were contacted to inform them of the objectives, methods, and use of data, and also receive their consent. The pertinent permissions were requested on an informed consent sheet addressed to the parents/guardians, and, before the tests were implemented, only students who had paternal authorization were permitted to participate. Second, the students were told that participation was voluntary and given the necessary instructions to fill out the questionnaire. They were also informed of the confidentiality and anonymity of data management. The study was approved by the Bioethics Committee of the University of Almería.

### 2.4. Data Analyses 

The SSPS version 23.0 for Windows (SPSS Inc., Chicago, IL, USA) was used for data processing and analysis. 

First, the descriptive analysis was done, and, in order to explore the relationships between variables, bivariate correlations were analyzed. Then, a stepwise multiple linear regression analysis was carried out with the aggression forms (overt proactive aggression, overt reactive aggression, relational proactive aggression, and relational reactive aggression) as the dependent variables. The predictor variables were emotional intelligence, values for positive adolescent development, and family functioning. Specifically, the variables that were correlated to the dependent variable were used to estimate the regression model in each case.

Lastly, a two-step cluster analysis was done to determine the profiles, using the variables that were included in each of the regression models. After the groups were classified based on the cluster solution, the means were compared using the Student’s *t* for independent samples to find out whether there were any differences between clusters with respect to types of aggression, and the Cohen’s *d* test was used to determine the effect size.

## 3. Results

### 3.1. Aggressive Conducts in Secondary Compulsory Education Students: Descriptive Analysis

Of the total sample, 13.6% (*n* = 43) had experienced or were currently suffering from violence by other students. On the contrary, 12.9% (*n* = 41) had used or were using some type of violence on other students, and 65.3% (*n* = 207) had seen violence used on other students. 

The gender distribution of the aggressors was 78% (*n* = 32) boys and 22% (*n* = 9) girls. In the group of victims, 55.8% (*n* = 24) were boys and 44.2% (*n* = 19) were girls.

The mean scores in the total sample for each of the dimensions of aggression were the following: overt proactive aggression (*M* = 0.24; *SD* = 0.37), overt reactive aggression (*M* = 0.55; *SD* = 0.54), relational proactive aggression (*M* = 0.22; *SD* = 0.35), and relational reactive aggression (*M* = 0.30; *SD* = 0.37). There were significant differences in over proactive aggression by sex (*t*_(315)_ = 3.36, *p* < 0.01, *d* = 0.38), where boys had higher scores (*M* = 0.30, *SD* = 0.43) than girls (*M* = 0.16, *SD* = 0.28). In relational proactive aggression, boys (*M* = 0.27, *SD* = 0.41) had significantly higher scores (*t*_(315)_ = 2.38, *p* < 0.05, *d* = 0.27) than girls (*M* = 0.18, *SD* = 0.28). 

In the group of aggressors, mean scores were significantly higher for all types of aggression (overt proactive aggression (*t*_(315)_ = 3.66, *p* < 0.01, *d* = 0.61), overt reactive aggression (*t*_(315)_ = 4.58, *p* < 0.001, *d* = 0.77), relational proactive aggression (*t*_(315)_ = 3.39, *p* < 0.01, *d* = 0.57), and Relational reactive aggression (*t*_(315)_ = 2.40, *p* < 0.05, *d* = 0.40)), compared to the group of nonaggressors. The group of victims had a significantly higher mean in overt reactive aggression (*t*_(315)_ = 1.99, *p* < 0.05, *d* = 0.33), than the group of nonvictims.

The age of participants was not correlated with any of the types of aggression analyzed. 

### 3.2. Emotional Intelligence, Values, and Family Functioning: Relationship with Aggression

The results derived from the correlation analysis, as shown in Table 1, indicate that Overt proactive aggression correlated negatively with most of the emotional intelligence factors (Intrapersonal: *r* = −0.13, *p* < 0.05, Interpersonal: *r* = −0.18, *p* < 0.01, Stress management: *r* = −0.20, *p* < 0.001, Mood: *r* = −0.15; *p* < 0.01), with Social values (*r* = −0.26, *p* < 0.001), Personal values (*r* = −0.26, *p* < 0.001), and Family functioning (*r* = −0.20, *p* < 0.001). Overt reactive aggression had negative correlations with stress management (*r* = −0.41, *p* < 0.001), Social values (*r* = −0.17, *p* < 0.01), Personal values (*r* = −0.15, *p* < 0.01), and Family functioning (*r* = −0.17, *p* < 0.01).

In relational proactive aggression, negative correlations were observed with some of the dimensions of emotional intelligence (Intrapersonal: *r* = −0.13, *p* < 0.05, interpersonal: *r* = −0.20, *p* < 0.001, stress management: *r* = −0.17, *p* < 0.01), social values (*r* = −0.24, *p* < 0.001), personal values (*r* = −0.24, *p* < 0.001), and family functioning (*r* = −0.18, *p* < 0.01).

Lastly, relational reactive aggression is negatively correlated with stress management (*r* = −0.22, *p*< 0.001), mood (*r* = −0.12, *p*< 0.05), social values (*r* = −0.12, *p* < 0.05), personal values (*r* = −0.17, *p*< 0.01), and family functioning (*r* = −0.12, *p* < 0.05). In general, the values of the correlations are between 0.2 to 0.4, which indicates a degree of statistical dependence among the weak variables.

Based on the results found in the correlation analyses, multiple regression models were constructed for each of the types of aggression, considering the variables in which correlations were detected in each case and entering them in the model as possible predictors.

### 3.3. Multiple Regression Model: Overt Proactive Aggression

As shown by the data presented in Table 2, the regression analysis found three models, where the third is the one with the most explanatory capacity, with 12.7% (*R*^2^ = 0.12) of the variance explained by the factors included in the model.

Independence of residuals was analyzed to confirm the validity of the model. The Durbin-Watson was *D* = 1.90, which confirms the absence of positive or negative self-correlation. The *t* is associated with a probability of error below 0.05 in all the variables included in the model (social values, stress management, and family functioning), while the standardized coefficients reveal that the variable with the most explanatory weight is social values. Lastly, absence of collinearity between the variables included in the model may be confirmed by the high tolerance and low VIF.

### 3.4. Multiple Regression Model: Overt Reactive Aggression

Table 3 shows the regression analysis for the three models in which the last explains 21.7% of the variance (*R*^2^ = 0.21). The Durbin-Watson *D* (*D* = 1.55) confirmed the absence of positive or negative self-correlation.

The *t* was associated with a probability of error below 0.05 for all the variables included in the model, and the standardized coefficients reveal that the variable with the most explanatory weight is stress management. Absence of collinearity may be assumed with tolerance and VIF near one.

### 3.5. Multiple Regression Model: Relational Proactive Aggression

The regression analysis of Relational proactive aggression, as shown in Table 4, resulted in four models, of which the fourth model had the most explanatory capacity with 12.2% (*R*^2^ = 0.12) of the variance explained by the factors included in the model.

To confirm model validity, independence of residuals was analyzed. The Durbin-Watson *D* was *D* = 1.96, which confirms the absence of positive or negative self-correlation. It was further observed that the *t* was associated with a probability of error below 0.05 in all the variables included in the model. In addition, the standardized coefficients revealed that the variables with the most explanatory weight were stress management and social values. Lastly, since tolerance was high and VIF was low, the absence of collinearity between variables may be assumed.

### 3.6. Multiple Regression Model: Relational Reactive Aggression

Table 5 shows the two models resulting from the regression analysis, of which the second model has an explained variance of 8.1% (*R*^2^ = 0.08). In this case, the Durbin-Watson *D* confirmed model validity (*D* = 1.91). The *t* test detected association with a probability of error below 0.05 for the variables included in the model: stress management and personal values. According to the standardized coefficients, stress management was the strongest predictor of relational reactive aggression. The *t* test showed an association with a probability of error less than 0.05 for the variables included in the model, stress management, and personal values. According to the standardized coefficients, stress management is the strongest predictor of Relational reactive aggression. In view of the tolerance and VIF, the absence of collinearity between variables may be assumed. 

### 3.7. Profiles According to Predictor Variables of Aggression and the Different Types of Aggression

**Figure 1:**
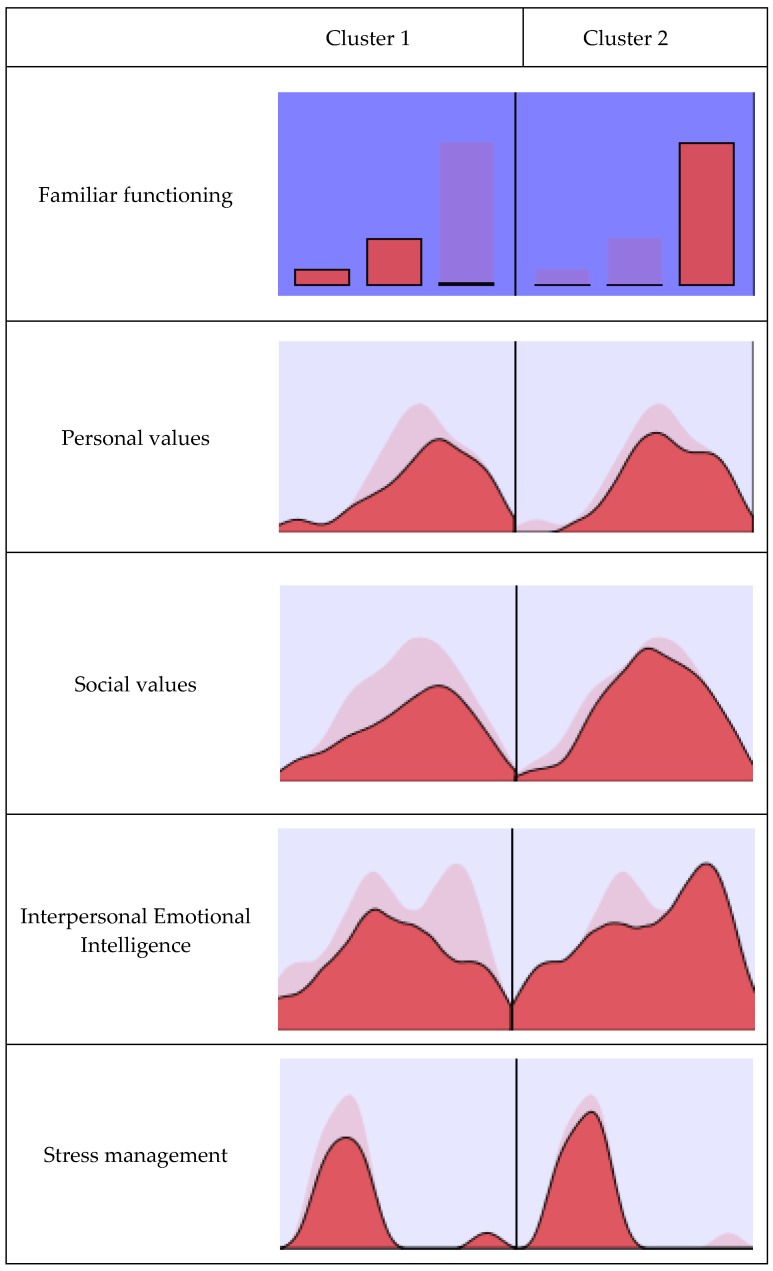
Cluster composition. Note: factors in order of importance of input.

A two-step cluster analysis was done with the variables that were included in the multiple linear regression models described above (family functioning, social values, personal values, stress management, and the interpersonal factor). For cluster construction, family functioning was taken as the categorical variable such that 0 to 3 points meant dysfunctional, 4 to 6 meant moderately functional, and 7 to 10 points was considered functional. In this case, family functioning is the predictor with the most relevance in cluster construction (Figure 1).

The inclusion of these variables resulted in two groups (Figure 1), with the following distribution: 30.6% (*n* = 97) of the participants in Cluster 1 and 69.4% (*n* = 220) in Cluster 2. Table 6 summarizes the mean scores on the variables analyzed in the total sample and for each of the clusters.

The first group resulting from the cluster analysis (Cluster 1) was characterized by low-moderate family functioning, mean scores below those found for the total sample in personal and social values, and similar to the sample mean interpersonal factor and stress management. While in the second cluster, with high family functioning, the mean scores were higher than the whole sample for personal and social values with scores near the sample mean in interpersonal and stress management.

After the groups were classified, based on the two-cluster solution, a Student’s t test for independent samples was done to find out whether there were any differences between clusters by type of aggression. The significant differences between the clusters were in Overt proactive aggression (*t*_(315)_ = 2.22, *p* < 0.05, *d* = 0.27), where Cluster 1 (*M* = 0.31, *DT* = 0.44) had higher scores than Cluster 2 (*M* = 0.20, *DT* = 0.33). No significant differences were observed for the rest of the types of aggression.

## 4. Discussion

Aggressive behavior is present in adolescence and its prevalence in recent years has been increasing [1,3]. The results of this study showed the percentages of students who have experienced or experience violence by their peers and those who have used violence on other students. Although these percentages are not too high, they are in consonance with those of Crespo-Ramos et al. [4] and may be due to the differences in the sample size. With regard to the sex of the aggressor, the percentage of boys is significantly higher than girls, which coincides with other studies where the highest percentages are for males [16,17]. On the contrary, in the study by Manring et al. [13] performed in primary school, the girls had higher scores. Nevertheless, in the group of victims, there were no significant differences between sexes.

The differences in scores by sex in the different types of aggression were only significant in Overt proactive aggression and Relational proactive aggression, where boys scored higher than girls in both. These results are in agreement with those found by Rieffe et al. [17] where boys had high percentages in both proactive and reactive aggression, but not with Van Hazebroek et al. [18] who found that it was reactive aggression where boys predominated, and no sex differences were found in proactive aggression. The results for the characterization of victim and aggressor, according to each of the aggression scales, showed that the group of aggressors had significantly higher means on all the scales than the nonaggressors. This coincides with other studies where the aggressors had Proactive aggressive behavior [19,20], while victims had higher scores in overt reactive aggression.

With regard to the correlation between the types of aggression and the emotional intelligence, values, and family functioning variables, we found a negative relationship, since, at higher levels of aggressiveness, emotional intelligence levels are lower [36,44], personal and social values are lower, and there is a higher risk of family dysfunction [42,46].

The multiple regression analysis showed that overt and relational proactive aggression were predicted or explained based on social values, stress management, and family functioning, including the interpersonal dimension in relational proactive aggression. In both overt and relational reactive aggression, the stress management and personal value dimensions stood out. Furthermore, family functioning was included in the first type. These results are in line with other studies where both overt and relational reactive aggression were characterized by low stress management and poor emotional regulation [32,36]. On the other hand, the analysis of family function and aggressive behavior supports the results recently obtained by Martínez et al. [43], where the family acts as a protective factor against the aggressive behavior of adolescents.

Lastly, this study found two profiles from the predictor variables (family functioning, personal values, social values, interpersonal, and stress management) and the differences in the type of aggression. Similar studies have profiled subjects by type of aggression [47]. 

## 5. Conclusions

These findings demonstrate the relationship between the variables studied and which dimensions have to be considered for intervention or in developing a tool since not all of them predicted each type of aggression. Nevertheless, the sample size should be pointed out as one of the limitations of this study. With a larger sample, future studies could be compared to find out whether the same variables explain aggressive behavior in adolescents. With regard to family functioning, it would also be of interest to find out whether the presence of aggressive behavior is due to antecedent family function or their consequence, since, at the present time, this is not clear in the literature. Regarding this variable, another limitation is detected related to the use of the scale and its validity, which is an issue that has been occasionally discussed by other authors [52], so the data obtained in the matter must be taken warily.

Lastly, highlight the practical implications of the results obtained, especially in the design of interventions aimed at the prevention of aggressive behavior, in all its forms. To this end, the relationships identified among the study variables are considered, which is why the implementation of programs based on the training of emotional intelligence or on the acquisition of values in adolescents is considered fundamental.

## Figures and Tables

**Table 1:** Correlations between types of aggression and emotional intelligence variables, values, and family functioning.

Variables	1	2	3	4	5	6	7	8	9	10	11	12
1. Overt proactive aggression	–											
2. Overt reactive aggression	0.61 ***	–										
3. Relational proactive aggression	0.79 ***	0.50 ***	–									
4. Relational reactive aggression	0.76 ***	0.58 ***	0.79 ***	–								
5. Intrapersonal	−0.13 *	−0.04	−0.13 *	−0.08	–							
6. Interpersonal	−0.18 **	0.19	−0.20 ***	−0.07	0.28 ***	–						
7. Stress management	−0.20 ***	−0.41 ***	−0.17 **	−0.22 ***	−0.00	−0.10	–					
8. Adaptability	−0.01	0.02	−0.05	−0.02	0.14**	0.36 ***	−0.09	–				
9. Mood	−0.15 **	−0.10	−0.08	−0.12 *	0.31 ***	0.19 ***	0.03	0.22 ***	–			
10. Social values	−0.26 ***	−0.17 **	−0.24 ***	−0.12 *	0.17 **	0.40 ***	0.05	0.23 ***	0.11 *	–		
11. Personal values	−0.26 ***	−0.15 **	−0.24 ***	−0.17 **	0.18 **	0.39 ***	−0.00	0.27 ***	0.06	0.74 ***	–	
12. Individualist values	0.02	0.08	0.02	0.09	0.15 **	0.18 **	−0.17 **	0.15 **	0.14 **	0.40 ***	0.47 ***	–
13. Family functioning	−0.20 ***	−0.17 **	−0.18 **	−0.12 *	0.19 ***	0.13 *	0.03	0.09	0.30 ***	0.20 ***	0.19 ***	0.18 **

* *p* < 0.05, ** *p* < 0.01, *** *p* < 0.001.

**Table 2:** Stepwise Multiple Linear Regression Model (overt proactive aggression).

Model	*R*	*R* ^2^	*R*^2^ Adjusted	Change Statistics					Durbin Watson
				SE	Change in *R*^2^	Change in *F*	Significance of the Change in *F*		
1	0.26	0.07	0.06	0.36	0.07	23.54	0.000		1.90
2	0.32	0.10	0.10	0.35	0.03	12.70	0.000		
3	0.35	0.12	0.11	0.35	0.02	7.72	0.006		
**Model 3**			**Unstandardized Coefficients**		**Standardized Coefficients**	***t***	**Sig.**	**Collinearity**	
			***B***	**SE**	**Beta**			**Tol.**	**VIF**
(Constant)			0.98	0.11		8.74	0.000		
Social values			−0.07	0.01	−0.22	−4.13	0.000	0.95	1.04
Stress management			−0.08	0.02	−0.18	−3.53	0.000	0.99	1.00
Family functioning			−0.02	0.00	−0.15	−2.77	0.006	0.95	1.04

**Table 3:** Stepwise Multiple Linear Regression Model (overt reactive aggression).

Model	*R*	*R* ^2^	*R*^2^ Adjusted	Change Statistics					Durbin Watson
				SE	Change in *R*^2^	Change in *F*	Sig. *F* Change		
1	0.41	0.17	0.17	0.49	0.17	66.81	0.000		1.55
2	0.44	0.20	0.19	0.49	0.02	9.83	0.002		
3	0.46	0.21	0.20	0.48	0.01	6.72	0.010		
**Model 3**			**Unstandardized Coefficients**		**Standardized Coefficients**	***t***	**Sig.**	**Collinearity**	
			***B***	**SE**	**Beta**			**Tol.**	**VIF**
(Constant)			1.85	0.17		10.37	0.000		
Stress management			−0.26	0.03	−0.41	−8.28	0.000	0.99	1.00
Family functioning			−0.03	0.01	−0.13	−2.59	0.010	0.96	1.04
Personal values			−0.07	0.02	−0.13	−2.59	0.010	0.96	1.04

**Table 4:** Stepwise Multiple Linear Regression Model (relational proactive aggression).

Model	*R*	*R* ^2^	*R*^2^ Corrected	Change Statistics					Durbin Watson
				SE	Change in *R*^2^	Change in *F*	Sig. *F* Change		
1	0.24	0.06	0.05	0.34	0.06	20.28	0.000		1.96
2	0.29	0.08	0.08	0.34	0.02	9.44	0.002		
3	0.32	0.10	0.09	0.34	0.01	6.72	0.010		
4	0.34	0.12	0.11	0.33	0.01	5.23	0.023		
**Model 4**			**Unstandardized Coefficients**		**Standardized Coefficients**	***t***	**Sig.**	**Collinearity**	
			***B***	**SE**	**Beta**			**Tol.**	**VIF**
(Constant)			1.04	0.12		8.08	0.000		
Social Values			−0.04	0.01	−0.15	−2.58	0.010	0.80	1.24
Stress management			−0.07	0.02	−0.18	−3.37	0.001	0.97	1.02
Interpersonal			−0.08	0.03	−0.14	−2.46	0.014	0.81	1.22
Family functioning			−0.01	0.00	−0.12	−2.28	0.023	0.95	1.04

**Table 5:** Stepwise Multiple Linear Regression Model (relational reactive aggression).

Model	*R*	*R* ^2^	*R*^2^ Adjusted	Change Statistics					Durbin Watson
				SE	Cambio en *R*^2^	Cambio en *F*	Sig. *F* Change		
1	0.22	0.05	0.04	0.36	0.05	16.40	0.000		1.91
2	0.28	0.08	0.07	0.36	0.03	10.69	0.001		
**Model 2**			**Unstandardized Coefficients**		**Standardized Coefficients**	***t***	**Sig.**	**Collinearity**	
			***B***	**SE**	**Beta**			**Tol.**	**VIF**
(Constant)			0.90	0.12		7.23	0.000		
Stress management			−0.09	0.02	−0.22	−4.13	0.000	1.00	1.00
Personal values			−0.06	0.02	−0.17	−3.27	0.001	1.00	1.00

**Table 6:** Mean scores for the total sample and clusters.

Variables	Total Sample (*N* = 317)	Cluster	
		1 (*n* = 97)	2 (*n* = 220)
Family functioning	*M* = 7.44 (*DT* = 2.32)	*M* = 4.51 (*DT* = 1.65)	*M* = 8.73 (*DT* = 1.05)
Personal values	*M* = 5.38 (*DT* = 1.02)	*M* = 5.15 (*DT* = 1.15)	*M* = 5.48 (*DT* = 0.94)
Social values	*M* = 4.89 (*DT* = 1.16)	*M* = 4.67 (*DT* = 1.26)	*M* = 4.98 (*DT* = 1.11)
Interpersonal Emotional Intelligence	*M* = 2.94 (*DT* = 0.59)	*M* = 2.89 (*DT* = 0.63)	*M* = 2.97 (*DT* = 0.58)
Stress management	*M* = 2.59 (*DT* = 0.85)	*M* = 2.63 (*DT* = 1.01)	*M* = 2.57 (*DT* = 0.78)
